# How human–AI collaboration event strength shapes information security behavior: evidence from a University in Shanghai

**DOI:** 10.3389/fpsyg.2026.1923587

**Published:** 2026-10-09

**Authors:** Wei Jin, Yuhui Ge

**Affiliations:** 1 Security Department (Armed Forces Department), Shanghai University of Electric Power, Shanghai, China; 2 Business School, University of Shanghai for Science and Technology, Shanghai, China

**Keywords:** cognitive overload, event system theory, human-AI collaboration, information security behavior, security vigilance

## Abstract

**Introduction:**

As artificial intelligence is increasingly integrated into higher education learning contexts, university students receive technological support while also facing new information security risks. However, existing research has paid limited attention to how the event characteristics of human-AI collaboration are associated with university students' information security behavior and to the psychological processes underlying these relationships. Drawing on event system theory, this study develops a dual pathway model to examine how human-AI collaboration event strength, reflected in event novelty, event disruption, and event criticality, is associated with information security behavior among university students through security vigilance and cognitive overload.

**Methods:**

Data were collected from students at a university in Shanghai using a three-wave time-lagged survey design, yielding 351 valid responses.

**Results:**

The results show that event novelty, event disruption, and event criticality are positively associated with security vigilance and cognitive overload. Security vigilance is positively associated with information security behavior, whereas cognitive overload is negatively associated with information security behavior. Further analyses indicate that these event strength attributes generate positive indirect effects through security vigilance and negative indirect effects through cognitive overload.

**Discussion:**

This study explains information security behavior during university students' use of artificial intelligence from an event-strength perspective and provides a more nuanced understanding of the underlying psychological processes. The findings provide a theoretical basis for universities to refine guidelines for artificial intelligence use, strengthen information security education, and optimize the design of human-AI collaborative learning.

## 1 Introduction

As artificial intelligence technologies continue to advance, their role in higher education is shifting from that of auxiliary tools to that of important technological agents that participate more deeply in learning processes (Crompton and Burke, 2023) The rise of generative artificial intelligence further accelerates this shift by enabling students to interact continuously with artificial intelligence in routine learning tasks and gradually develop more established forms of human–AI collaboration (Atchley et al., 2024; Chan and Hu, 2023). At the same time, the use of artificial intelligence raises concerns regarding content reliability, privacy protection, and institutional regulation (Bond et al., 2024; Cotton et al., 2024; García-López et al., 2025; Kasneci et al., 2023; Tlili et al., 2023). Although universities have begun to formulate relevant policies and instructional guidelines, limitations remain in data privacy protection, appropriate use, and classroom guidance (Jin et al., 2025; McDonald et al., 2025). Accordingly, information security behavior among university students has become an important issue that requires greater attention as human–AI collaboration becomes increasingly embedded in higher education.

Information security behavior generally refers to the behavioral choices adopted by individuals to protect information assets, avoid potential risks, and comply with relevant security norms during the acquisition, processing, storage, transmission, and sharing of information. In information security research, this concept is commonly understood as a set of security practices and compliance behaviors intended to protect the confidentiality, integrity, and availability of information (Lebek et al., 2014). In higher education contexts, university students are frequent users of artificial intelligence technologies and are also directly exposed to information security risks. Their information security behavior affects the protection of personal privacy, account security, and academic materials, as well as the broader security of digital learning environments on university campuses. Existing research on artificial intelligence in higher education has developed along several lines. First, studies have examined the potential value of artificial intelligence for learning support, personalized learning, instructional assistance, feedback, and assessment (Naznin et al., 2025; Ogunleye et al., 2024; Wang et al., 2025; Zawacki-Richter et al., 2019). Second, increasing attention has been paid to the risks and challenges associated with artificial intelligence use, including academic integrity, data privacy, ethical responsibility, algorithmic bias, and governance frameworks (Bittle and El-Gayar, 2025; Bond et al., 2024). Third, as generative artificial intelligence becomes more widely used in education, greater attention has been directed toward institutional preparedness, particularly the development of university guidelines, course rules, training mechanisms, and assessment reforms (Jin et al., 2025; McDonald et al., 2025). Overall, prior research has provided substantial insight into the applications, benefits, and governance challenges of artificial intelligence in higher education. However, limited attention has been paid to information security behavior among university students in human–AI collaboration contexts and to the psychological mechanisms underlying such behavior. Students may respond differently when artificial intelligence is incorporated into similar learning tasks. Some students may overlook potential risks and reduce information verification, whereas others may adopt more cautious and protective approaches. A more theoretically grounded framework is therefore needed to explain these differences.

Event system theory provides an appropriate perspective for addressing this issue. The theory proposes that changes in individual attitudes and behaviors are influenced by the extent to which contextual factors are perceived as events with sufficient strength. Event strength is reflected in three dimensions: event novelty, event disruption, and event criticality. Event novelty captures the extent to which an event differs from prior experience. Event disruption reflects the extent to which an event interrupts existing task processes, cognitive structures, and behavioral patterns. Event criticality captures the extent to which an event is associated with goal attainment and consequential outcomes (Morgeson et al., 2015). Human–AI collaboration can therefore be conceptualized as a dynamic event that is embedded in learning activities and shapes how university students process information, complete tasks, and evaluate potential risks. Applying event system theory to human–AI collaboration can provide a more precise explanation of why students differ in their information security behavior when artificial intelligence is incorporated into learning activities.

From this theoretical perspective, human–AI collaboration event strength may influence information security behavior through two psychological mechanisms: cognitive overload and security vigilance. Stronger human–AI collaboration events may increase cognitive overload. According to event system theory, events characterized by greater novelty, disruption, and criticality are more likely to elicit cognitive and behavioral responses (Morgeson et al., 2015). When human–AI collaboration involves greater event novelty, students need to understand unfamiliar tools, evaluate new sources of information, and adjust their existing approaches within a limited period. When event disruption is greater, established learning processes and judgment patterns are interrupted, requiring additional cognitive resources for adaptation. When event criticality is greater, pressure associated with task completion and expected outcomes further limits the cognitive resources available for risk recognition and security related judgment. Under these conditions, students may prioritize efficiency and task completion, thereby paying less attention to privacy protection, permission settings, and content verification. Their information security behavior may consequently decline.

Human–AI collaboration event strength may also promote information security behavior by increasing security vigilance. Event system theory suggests that stronger events can interrupt habitual cognition, redirect attention, and increase sensitivity to potential risks and behavioral consequences (Morgeson et al., 2015). When students perceive greater event novelty, event disruption, or event criticality, more attention may be directed toward security risks arising during information processing. Students may become more cautious about whether uploaded data contain sensitive information, whether external platforms are trustworthy, whether artificial intelligence outputs are reliable, and whether requested permissions are appropriate. The heightened visibility of these risks can increase security vigilance and encourage more cautious information processing and protective behavior. Human–AI collaboration event strength may therefore generate positive behavioral effects by increasing sensitivity to potential risks.

This study makes three contributions. First, this study situates information security behavior research in the campus context where university students use AI to assist with learning tasks, with particular attention to students' information security behavior under human–AI collaboration. In doing so, it broadens the contextual foundation for understanding information security behavior in AI-use settings. Second, by conceptualizing human–AI collaboration as an event and applying event system theory to characterize its strength in terms of event novelty, disruption, and criticality, this study provides a new theoretical lens for explaining variations in university students' information security behavior during AI use and further refines the application of event system theory in human–AI collaboration research. Third, this study simultaneously incorporates security vigilance and cognitive overload as two psychological mechanisms, explaining how human–AI collaboration event strength is linked to information security behavior through distinct cognitive responses. This broadens understanding of the psychological mechanisms underlying information security behavior during AI use and provides a more comprehensive explanatory framework for understanding the complex formation of information security behavior in human–AI collaboration contexts. The findings provide a theoretical basis for universities to refine AI use guidelines, strengthen information security education, and optimize the design of AI-assisted learning tasks. The theoretical model is presented in Figure 1.

**Figure 1:**
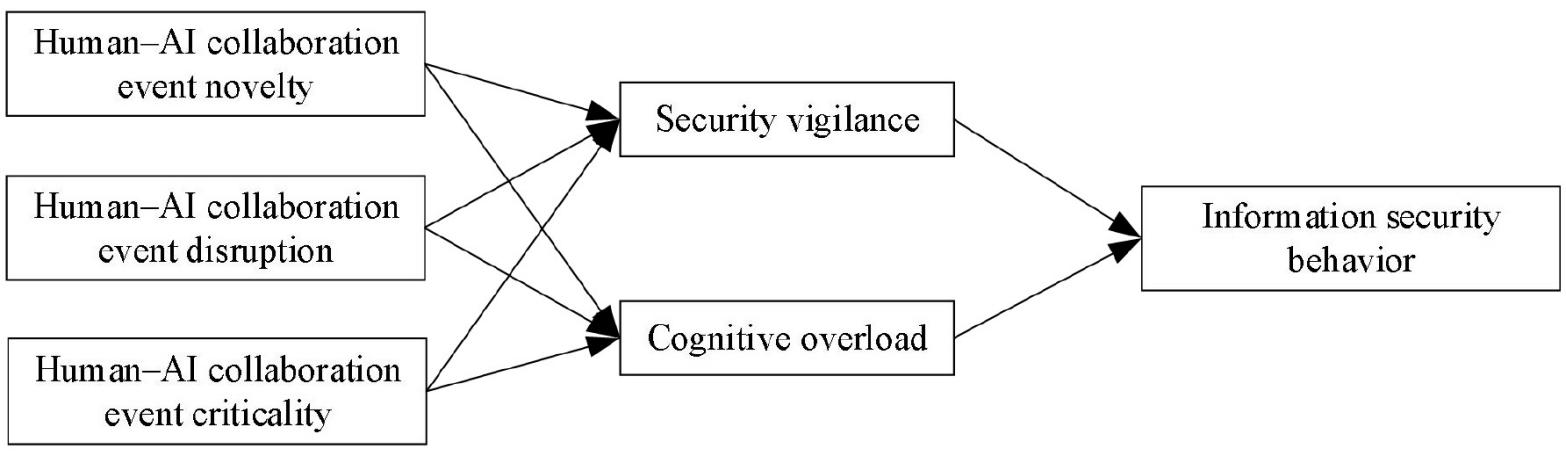
Theoretical model.

## 2 Theoretical analysis and hypothesis development

### 2.1 Event system theory

In the context of this study, collaboration between university students and artificial intelligence can be viewed as a dynamic event embedded in learning activities. Event system theory offers an appropriate theoretical perspective for explaining how human–AI collaboration event strength influences students' cognitive and behavioral responses. The theory was proposed by Morgeson et al. (2015) to explain how events influence entities. The theory conceptualizes events as external dynamic experiences encountered by entities. Events constitute part of environmental change, and their effects are shaped by temporal and spatial conditions. The core attributes of an event include time, space, and strength. Among these attributes, event strength plays a central role in determining the extent to which an event influences an entity. Event strength is reflected in three dimensions: event novelty, event disruption, and event criticality.

Various events may arise during ongoing activities, but their influence depends on the extent to which they are perceived as sufficiently strong. Event novelty reflects the extent to which an event differs from prior experience and is perceived as unexpected. When an event is highly novel, individuals often lack established procedures or prior experience that can be directly applied. Additional information processing is therefore required to understand the event and adapt to its consequences. Event disruption refers to the extent to which an event interrupts established activities and routines. Greater disruption requires individuals to adjust their existing behavioral patterns in response to changes in the environment. Event criticality reflects the extent to which an event is prioritized because of its relevance to individual goals and outcomes. More critical events require greater attention because they have stronger implications for goal attainment (Morgeson et al., 2015). The basic framework of event system theory is shown in Figure 2.

**Figure 2:**
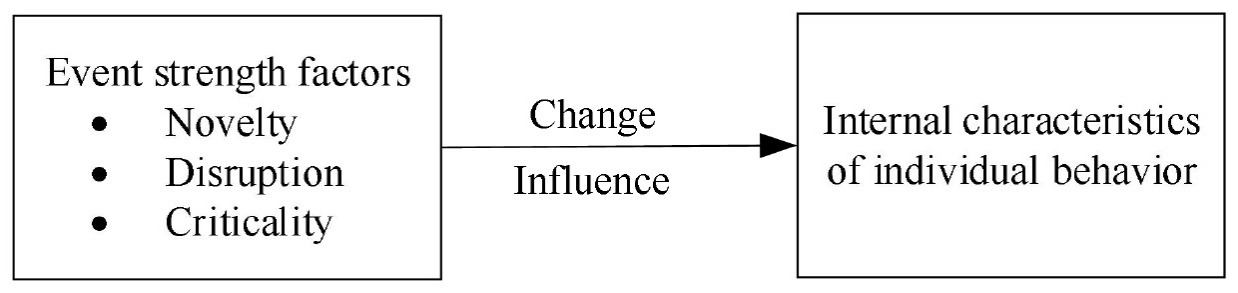
Event system theory model.

Research based on event system theory suggests that event strength can trigger responses within entities and influence subsequent outcomes. The theory has been supported by empirical studies conducted at different levels of analysis (Liu et al., 2023) At the individual and team levels, interruptive events have been found to facilitate knowledge transfer and contribute to the development of new procedures and routines (Zellmer-Bruhn, 2003). Research on team events further shows that leaders can improve team performance through proactive intervention under specific event conditions (Morgeson, 2005). Event criticality and duration also shape the extent to which disruptive events interfere with routine team functioning (Morgeson and DeRue, 2006). At the organizational level, natural disasters have been categorized according to the extent of property damage, and events of different strengths have been shown to influence charitable giving (Tilcsik and Marquis, 2013). Based on the theoretical framework above, the following section develops specific hypotheses concerning the relationships among human–AI collaboration event strength, security vigilance, cognitive overload, and information security behavior.

### 2.2 Hypothesis development

In human–AI collaborative learning contexts, event novelty, event disruption, and event criticality may influence security vigilance through their effects on students' attention to security-related cues. Event system theory suggests that stronger events are more likely to attract attention and prompt individuals to reassess the current situation and their existing responses (Morgeson et al., 2015). Information security research similarly shows that attention to potential risks contributes to more cautious information processing and protective behavior (Bulgurcu et al., 2010; Lebek et al., 2014; Torten et al., 2018; Tsai et al., 2016; van Bavel et al., 2019). On this basis, the three dimensions of event strength may enhance security vigilance through distinct mechanisms.

When human–AI collaboration differs substantially from students' prior learning experience, established judgment patterns and prior experience become less directly applicable, requiring students to reassess the reliability of artificial intelligence tools and their outputs. Prior research shows that university students using generative artificial intelligence for learning attend to content accuracy, privacy risks, and ethical concerns (Chan and Hu, 2023) and need to maintain reflective judgment rather than accept artificial intelligence outputs without evaluation (Atchley et al., 2024). Information security research also indicates that the ability to recognize potential threats contributes to risk avoidance (Arachchilage and Love, 2014). Therefore, greater event novelty is expected to increase students' attention to uncertainty and potential risks, thereby strengthening security vigilance.

When artificial intelligence substantially changes established approaches to information acquisition, task completion, and content verification, existing learning routines no longer support routine judgment, requiring students to reassess both the learning process and artificial intelligence outputs. Event system theory suggests that disruptive events interrupt established routines and require individuals to adjust their responses (Morgeson et al., 2015; Morgeson and DeRue, 2006). Hwang and Lee (2025) show that students engaged in human–AI collaborative content creation often revise and refine artificial intelligence outputs rather than accept them directly. Therefore, greater event disruption is expected to increase the need to reevaluate information and attend to potential risks, thereby strengthening security vigilance.

When human–AI collaborative tasks are closely tied to course grades, academic work, or other consequential learning outcomes, the potential costs of artificial intelligence errors or misuse become more salient. Event system theory suggests that critical events receive greater attention because of their relevance to goal attainment (Morgeson et al., 2015). Chan and Hu (2023) also show that students recognize the learning value of artificial intelligence while remaining concerned about its accuracy and privacy implications. Therefore, greater event criticality is expected to increase students' attention to the potential consequences of artificial intelligence use, thereby strengthening security vigilance. Based on the preceding arguments, the following hypotheses are proposed:

*H1a: Human–AI collaboration event novelty is positively associated with security vigilance*.

*H1b: Human–AI collaboration event disruption is positively associated with security vigilance*.

*H1c: Human–AI collaboration event criticality is positively associated with security vigilance*.

In human–AI collaborative learning contexts, event novelty, event disruption, and event criticality may increase cognitive overload by increasing students' information-processing and adaptation demands. Cognitive load theory suggests that working memory resources are limited and that cognitive overload becomes more likely when task demands exceed available cognitive resources (Sweller, 2023). Although artificial intelligence can provide learning support, students still need to understand and evaluate generated content and its fit with task requirements (García-López et al., 2025), while verifying and adjusting artificial intelligence outputs may create additional cognitive demands (Patac and Patac, 2025). On this basis, the three dimensions of event strength may increase cognitive overload through different types of cognitive demands.

When human–AI collaboration differs substantially from students' established learning experience, existing knowledge structures and response patterns become less directly applicable, requiring students to devote additional cognitive resources to understanding artificial intelligence tools and developing new criteria for judgment. Event system theory suggests that novel events often lack established response procedures and therefore require more intensive information processing (Morgeson et al., 2015). Cognitive load research similarly indicates that unfamiliar problems require individuals to process more information elements within working memory (Chen et al., 2023). Yan et al. (2024) further note that effective use of generative artificial intelligence requires adaptive capacity. Therefore, greater event novelty is expected to increase the cognitive resources required to understand and adapt to new forms of human–AI collaboration, thereby increasing the likelihood of cognitive overload.

When artificial intelligence substantially changes established learning processes, existing task routines become less effective in conserving cognitive resources, requiring students to reorganize how information is acquired, tasks are completed, and artificial intelligence outputs are verified. Event system theory suggests that disruptive events interrupt established routines and require behavioral adjustment (Morgeson et al., 2015; Morgeson and DeRue, 2006). Tian and Zhang (2025) also find that dependence on artificial intelligence increases cognitive fatigue. Therefore, greater event disruption is expected to increase the cognitive resources required to reorganize and continuously monitor learning processes, thereby increasing the likelihood of cognitive overload.

When human–AI collaborative tasks are associated with more important learning goals and outcomes, students need to devote greater cognitive resources to ensuring the quality of artificial intelligence outputs and task completion. Event system theory suggests that critical events receive greater processing priority because of their relevance to goal attainment (Morgeson et al., 2015). Patac and Patac (2025) also indicate that artificial intelligence assisted learning requires individuals to maintain independent judgment despite potential efficiency gains. Therefore, greater event criticality is expected to increase sustained cognitive investment in verification and quality control, thereby increasing the likelihood of cognitive overload. Based on the preceding arguments, the following hypotheses are proposed:

*H2a: Human–AI collaboration event novelty is positively associated with cognitive overload*.

*H2b: Human–AI collaboration event disruption is positively associated with cognitive overload*.

*H2c: Human–AI collaboration event criticality is positively associated with cognitive overload*.

Security vigilance reflects a psychological state in which individuals remain sensitive to potential information security risks and actively evaluate information content and the consequences of their actions. In human–AI collaborative learning contexts, such vigilance is reflected in the continued evaluation of artificial intelligence generated content before it is accepted or used. Wang and Zhang, (2026) show that collaboration with artificial intelligence encourages individuals to systematically evaluate potential errors, reasoning processes, and information sources in generated content. Information security research also identifies awareness of and attention to security issues as important foundations of information security behavior (Lebek et al., 2014; Torten et al., 2018). Therefore, greater security vigilance is expected to increase students' attention to potential risks and encourage more cautious handling of artificial intelligence generated information, thereby strengthening information security behavior. Based on the preceding arguments, the following hypothesis is proposed:

*H3: Security vigilance is positively associated with information security behavior among university students*.

Cognitive overload refers to a state in which cognitive demands exceed the resources available to an individual, reducing the efficiency of information processing and the quality of judgment (Chen et al., 2023; Sweller, 2023). Information security behavior typically requires individuals to allocate attention and continuously evaluate potential risks. When cognitive resources are heavily occupied, individuals are more likely to concentrate their attention on the immediate task, leaving fewer resources available for security-related judgments (Ganye and Smith, 2024). Jenkins et al. (2021) also find that greater effort requirements weaken the translation of security intentions into actual behavior. In human–AI collaborative learning contexts, cognitive overload may direct students' attention toward task completion while reducing the resources available for verifying artificial intelligence generated content and potential security risks. Therefore, greater cognitive overload is expected to constrain the cognitive resources required for cautious information processing, thereby reducing information security behavior. Based on the preceding arguments, the following hypothesis is proposed:

*H4: Cognitive overload is negatively associated with information security behavior among university students*.

The preceding arguments indicate that human–AI collaboration event novelty, disruption, and criticality can increase students' security vigilance, which in turn promotes more cautious information processing and stronger information security behavior. By directing greater attention to potential security risks, security vigilance therefore provides the psychological link between human–AI collaboration event strength and information security behavior. Based on the preceding arguments, the following hypotheses are proposed:

*H5a: Security vigilance mediates the relationship between human–AI collaboration event novelty and information security behavior among university students*.

*H5b: Security vigilance mediates the relationship between human–AI collaboration event disruption and information security behavior among university students*.

*H5c: Security vigilance mediates the relationship between human–AI collaboration event criticality and information security behavior among university students*.

The preceding arguments indicate that human–AI collaboration event novelty, disruption, and criticality can increase students' information-processing and cognitive demands, thereby heightening cognitive overload. Cognitive overload, in turn, reduces the cognitive resources available for cautious information processing and weakens information security behavior. Cognitive overload therefore provides the psychological link through which stronger human–AI collaboration events can undermine information security behavior. Based on the preceding arguments, the following hypotheses are proposed:

*H6a: Cognitive overload mediates the relationship between human–AI collaboration event novelty and information security behavior among university students*.

*H6b: Cognitive overload mediates the relationship between human–AI collaboration event disruption and information security behavior among university students*.

*H6c: Cognitive overload mediates the relationship between human–AI collaboration event criticality and information security behavior among university students*.

## 3 Research method

### 3.1 Data collection and sample characteristics

This study focuses on students at a university in Shanghai. The university is a multidisciplinary institution with a diverse student body across science, engineering, humanities, and social sciences. This diversity allows the study to obtain information on AI use patterns and information security behaviors among students from different academic backgrounds, providing an appropriate sample base for the present study. Questionnaires were distributed through internal university channels and covered students from different majors and year levels. Participation was voluntary and anonymous. To reduce potential common method bias associated with data collection at a single time point, a three-wave time-lagged survey design was adopted, with a 4-week interval between waves.

In the first wave, questionnaires were distributed to 650 students. Demographic information, frequency of generative artificial intelligence use, event novelty, event disruption, and event criticality were measured. A total of 514 valid responses were collected, resulting in a valid response rate of 79.08%. 4 weeks later, the second wave questionnaire was distributed to the 514 respondents who had completed the first wave. Security vigilance and cognitive overload were measured. A total of 458 valid responses were collected, resulting in a valid response rate of 89.11%. After another 4–week interval, the third wave questionnaire was distributed to the 458 respondents who had completed the second wave. Information security behavior was measured. A total of 351 valid responses were collected, resulting in a valid response rate of 76.64%.

The sample characteristics are presented in Table 1. The final sample includes 183 male respondents, accounting for 52.14% of the sample, and 168 female respondents, accounting for 47.86%. Respondents aged 18 to 20 and 21 to 23 account for the largest proportions of the sample, at 37.04% and 44.16%, respectively. Senior students represent the largest academic level group, accounting for 28.77% of the sample. Students majoring in humanities and social sciences account for the largest proportion, at 31.62%, followed by students majoring in science and engineering, at 28.21%. Regarding the frequency of generative artificial intelligence use, 44.44% of respondents report using generative artificial intelligence once per day, while 29.34% report using it several times per day.

**Table 1:** Sample characteristics.

Variable	Category	Frequency	Percentage (%)
Gender	Male	183	52.14
	Female	168	47.86
Age	18 and under	36	10.26
	18–20	130	37.04
	21–23	155	44.16
	24 and above	30	8.55
Year of study	Freshman	70	19.94
	Sophomore	71	20.23
	Junior	79	22.51
	Senior	101	28.77
	Postgraduate and above	30	8.55
Major type	Science & Engineering	99	28.21
	Humanities & Social sciences	111	31.62
	Arts & Physical education	52	14.81
	Medicine	72	20.51
	Others	17	4.84
Frequency of generative AI use	Multiple times per day	103	29.34
	Once per day	156	44.44
	Several times per week	53	15.10
	Less often	39	11.11

*N* = 351.

### 3.2 Measures

Except for the demographic variables, all constructs were assessed using established scales adapted to the context of human–AI collaborative learning among university students. All items were rated on a five-point Likert scale ranging from 1 = strongly disagree to 5 = strongly agree. The complete measurement items are provided in Appendix A.

#### 3.2.1 Human–AI collaboration event strength

Based on the event system theory framework proposed by Morgeson et al. (2015), this construct comprises event novelty, event disruption, and event criticality. Event novelty was assessed with four items, including “The way I complete learning tasks in collaboration with AI is novel to me” (Cronbach's alpha = 0.878). Event disruption was assessed with three items, including “The involvement of AI changes my original learning process” (Cronbach's alpha = 0.811). Event criticality was assessed with four items, including “Learning tasks completed in collaboration with AI have an important impact on my course grades” (Cronbach's alpha = 0.877).

#### 3.2.2 Security vigilance

The six-item scale was adapted from Wang and Zhang (2026). A sample item is “I critically evaluate the accuracy of content generated by AI” (Cronbach's alpha = 0.901).

#### 3.2.3 Cognitive overload

The ten-item scale was adapted from Leppink et al. (2013). A sample item is “The instructions or explanations provided in the learning task contain many unclear expressions” (Cronbach's alpha = 0.931).

#### 3.2.4 Information security behavior

The three-item scale was adapted from Tang et al. (2021). A sample item is “I expect that I will think twice before clicking unknown links recommended by generative artificial intelligence or downloading unknown files” (Cronbach's alpha = 0.855).

#### 3.2.5 Control variables

Following Spector and Brannick (2011), gender, academic level, field of study, and frequency of generative artificial intelligence use were included as control variables because individual characteristics and technology use may influence the results.

## 4 Results

### 4.1 Reliability and validity

To assess the reliability and convergent validity of the measures, standardized factor loadings, average variance extracted, composite reliability, and Cronbach's alpha coefficients are calculated. As shown in Table 2, the standardized factor loadings range from 0.723 to 0.832, exceeding the recommended threshold of 0.70. The average variance extracted values range from 0.575 to 0.663, the composite reliability values range from 0.812 to 0.931, and the Cronbach's alpha coefficients range from 0.811 to 0.931. These results indicate that all constructs demonstrate satisfactory reliability and convergent validity.

**Table 2:** Reliability and convergent validity.

Variable	Code	Item	Standardized factor loading	AVE	CR	Cronbach's α
Human-AI collaboration novelty	HACN	HACN1	0.803	0.643	0.878	0.878
		HACN2	0.773			
		HACN3	0.805			
		HACN4	0.826			
Human-AI collaboration disruption	HACD	HACD1	0.801	0.590	0.812	0.811
		HACD2	0.779			
		HACD3	0.723			
Human-AI collaboration criticality	HACC	HACC1	0.755	0.641	0.877	0.877
		HACC2	0.810			
		HACC3	0.819			
		HACC4	0.816			
Security vigilance	SV	SV1	0.795	0.604	0.901	0.901
		SV2	0.776			
		SV3	0.806			
		SV4	0.758			
		SV5	0.745			
		SV6	0.781			
Cognitive overload	CO	CO1	0.769	0.575	0.931	0.931
		CO2	0.761			
		CO3	0.733			
		CO4	0.732			
		CO5	0.747			
		CO6	0.785			
		CO7	0.756			
		CO8	0.796			
		CO9	0.769			
		CO10	0.735			
Information security behavior	ISB	ISB1	0.832	0.663	0.855	0.855
		ISB2	0.827			
		ISB3	0.782			

Discriminant validity is further assessed using the Fornell–Larcker criterion and the heterotrait–monotrait ratio (HTMT). As shown in Table 3, the square roots of the AVE are reported on the diagonal, and each value exceeds the corresponding inter-construct correlations reported in the correlation matrix, satisfying the Fornell–Larcker criterion. All HTMT values are below the recommended threshold of 0.85. These results indicate satisfactory discriminant validity among the six constructs.

**Table 3:** Discriminant validity.

Variable	1	2	3	4	5	6
1. HACN	0.802					
2. HACD	0.567	0.768				
3. HACC	0.474	0.532	0.801			
4. SV	0.419	0.448	0.454	0.777		
5. CO	0.375	0.403	0.363	0.200	0.759	
6. ISB	0.227	0.287	0.304	0.337	0.269	0.814

*N* = 351. HACN, human–AI collaboration event novelty; HACD, human–AI collaboration event disruption; HACC, human–AI collaboration event criticality; SV, security vigilance; CO, cognitive overload; ISB, information security behavior. Diagonal entries are the square roots of AVE; entries below the diagonal are HTMT ratios.

### 4.2 Confirmatory factor analysis

A confirmatory factor analysis is conducted to evaluate the proposed six-factor measurement structure comprising human–AI collaboration event novelty, human–AI collaboration event disruption, human–AI collaboration event criticality, security vigilance, cognitive overload, and information security behavior. The six-factor model is compared with alternative five-factor, four-factor, three-factor, two-factor, and single-factor models. As shown in Table 4, the six-factor model demonstrates a satisfactory fit to the data: χ^2^ = 408.250, df = 390, χ^2^/df = 1.047, CFI = 0.997, TLI = 0.996, SRMR = 0.029, and RMSEA = 0.012. The six-factor model also fits the data substantially better than the alternative models.

**Table 4:** Results of confirmatory factor analysis.

Model	Factor structure	*χ^2^/*df	CFI	TLI	SRMR	RMSEA
Seven-factor model	HACN, HACD, HACC, SV, CO, ISB, CMV	1.059	0.996	0.995	0.032	0.013
Six-factor model	HACN, HACD, HACC, SV, CO, ISB	1.047	0.997	0.996	0.029	0.012
Five-factor model	HACN + HACD, HACC, SV, CO, ISB	1.646	0.955	0.951	0.048	0.043
Four-factor model	HACN + HACD + HACC, SV, CO, ISB	2.702	0.880	0.870	0.055	0.070
Three-factor model	HACN + HACD + HACC, SV + CO, ISB	5.565	0.677	0.651	0.144	0.114
Two-factor model	HACN + HACD + HACC, SV + CO + ISB	6.822	0.586	0.554	0.164	0.129
Single-factor model	HACN + HACD + HACC + SV + CO + ISB	8.753	0.448	0.407	0.173	0.149

HACN, human–AI collaboration event novelty; HACD, human–AI collaboration event disruption; HACC, human–AI collaboration event criticality; SV, security vigilance; CO, cognitive overload; ISB, information security behavior. The plus sign indicates that the corresponding constructs are combined into a single factor.

### 4.3 Common method bias

Because the data are collected from self-reported questionnaires, common method bias may be present. Harman's single-factor test and a common method factor test are therefore conducted to assess whether common method bias substantially affects the results. First, all measurement items are included in an unrotated exploratory factor analysis. The results show that the first factor accounts for 30.067% of the total variance, which is below the threshold of 40%. Second, a common method factor is added to the six-factor model to construct a seven-factor model. The fit indices of the two models are then compared. As shown in Table 4, the addition of the common method factor does not improve model fit substantially (ΔCFI = −0.001, ΔTLI = −0.001, ΔSRMR = 0.003, and ΔRMSEA = 0.001). Taken together, these results indicate that common method bias does not pose a serious threat to the findings.

### 4.4 Descriptive statistics and correlations

Table 5 presents the means, standard deviations, and correlations among the variables. Human–AI collaboration event novelty is positively correlated with security vigilance (r = 0.373, *p* < 0.001) and cognitive overload (r = 0.339, *p* < 0.001). Human–AI collaboration event disruption is positively correlated with security vigilance (r = 0.384, *p* < 0.001) and cognitive overload (r = 0.350, *p* < 0.001). Human–AI collaboration event criticality is positively correlated with security vigilance (r = 0.404, *p* < 0.001) and cognitive overload (r = 0.328, *p* < 0.001). Security vigilance is positively correlated with information security behavior (r = 0.295, *p* < 0.001), whereas cognitive overload is negatively correlated with information security behavior (r = −0.240, *p* < 0.001). These results are generally consistent with the theoretical expectations and provide preliminary support for the proposed hypotheses.

**Table 5:** Descriptive statistics and correlations.

Variable	Mean	SD	1	2	3	4	5	6	7	8	9	10
1. Gender	1.479	0.500										
2. Age	2.510	0.792	0.010									
3. Grade	2.858	1.270	0.018	0.904^***^								
4. AIUSE	2.080	0.941	0.028	−0.105	−0.067							
5. HACN	3.269	1.047	−0.009	−0.005	−0.021	−0.055	(0.878)					
6. HACD	3.255	1.048	−0.006	−0.044	−0.075	−0.050	0.479^***^	(0.811)				
7. HACC	3.375	1.031	−0.129^*^	−0.065	−0.075	0.018	0.415^***^	0.449^***^	(0.877)			
8. SV	3.328	0.948	−0.074	−0.049	−0.037	−0.053	0.373^***^	0.384^***^	0.404^***^	(0.901)		
9. CO	2.981	0.894	0.020	−0.125^*^	−0.090	0.045	0.339^***^	0.350^***^	0.328^***^	0.183^***^	(0.931)	
10. ISB	3.351	1.081	−0.030	−0.062	−0.052	−0.019	0.197^***^	0.239^***^	0.263^***^	0.295^***^	−0.240^***^	(0.855)

*N* = 351. AIUSE, frequency of generative artificial intelligence use; HACN, human–AI collaboration event novelty; HACD, human–AI collaboration event disruption; HACC, human–AI collaboration event criticality; SV, security vigilance; CO, cognitive overload; ISB, information security behavior. Cronbach's alpha coefficients are reported in parentheses on the diagonal. ^*^p < 0.05. ^***^p < 0.001.

### 4.5 Hypothesis testing

#### 4.5.1 . Direct effects

Gender, academic level, field of study, and frequency of generative artificial intelligence use are included as control variables. The results of the direct effect tests are reported in Table 6. Human–AI collaboration event novelty is positively associated with security vigilance (β = 0.184, *p* < 0.01). Human–AI collaboration event disruption is positively associated with security vigilance (β = 0.187, *p* < 0.01). Human–AI collaboration event criticality is also positively associated with security vigilance (β = 0.239, *p* < 0.001). Thus, H1a, H1b, and H1c are supported. Human–AI collaboration event novelty is positively associated with cognitive overload (β = 0.187, *p* < 0.01). Human–AI collaboration event disruption is positively associated with cognitive overload (β = 0.182, *p* < 0.01). Human–AI collaboration event criticality is also positively associated with cognitive overload (β = 0.170, *p* < 0.01). Thus, H2a, H2b, and H2c are supported. Security vigilance is positively associated with information security behavior (β = 0.185, *p* < 0.001), whereas cognitive overload is negatively associated with information security behavior (β = −0.447, *p* < 0.001). Thus, H3 and H4 are supported.

**Table 6:** Results of direct effect tests.

Hypothesis	Path	β	Standard error	t value	*p* value	f^2^	R^2^	Result
H1a	HACN → SV	0.184	0.056	3.294	< 0.01	0.032	0.244	Supported
H1b	HACD → SV	0.187	0.057	3.283	< 0.01	0.032		Supported
H1c	HACC → SV	0.239	0.055	4.314	< 0.001	0.055		Supported
H2a	HACN → CO	0.187	0.058	3.251	< 0.01	0.031	0.200	Supported
H2b	HACD → CO	0.182	0.059	3.100	< 0.01	0.028		Supported
H2c	HACC → CO	0.170	0.057	2.976	< 0.01	0.026		Supported
H3	SV → ISB	0.185	0.053	3.490	< 0.001	0.036	0.284	Supported
H4	CO → ISB	−0.447	0.052	−8.674	< 0.001	0.223		Supported

*N* = 351. β denotes the standardized regression coefficient. HACN denotes human–AI collaboration event novelty. HACD denotes human–AI collaboration event disruption. HACC denotes human–AI collaboration event criticality. SV denotes security vigilance. CO denotes cognitive overload. ISB denotes information security behavior.

#### 4.5.2 . Indirect effects

The effects are tested using 5,000 bootstrap resamples. The direct effects of human–AI collaboration event novelty, event disruption, and event criticality on information security behavior are 0.110, 0.173, and 0.210, respectively, while their corresponding total effects are 0.061, 0.127, and 0.179. The indirect effects through security vigilance and cognitive overload are presented in Table 7. Human–AI collaboration event novelty has a significant positive indirect effect on information security behavior through security vigilance. The indirect effect is 0.034, with a 95% bootstrap confidence interval of [0.009, 0.068]. Human–AI collaboration event disruption has a significant positive indirect effect on information security behavior through security vigilance. The indirect effect is 0.035, with a 95% bootstrap confidence interval of [0.009, 0.070]. Human–AI collaboration event criticality also has a significant positive indirect effect on information security behavior through security vigilance. The indirect effect is 0.044, with a 95% bootstrap confidence interval of [0.015, 0.084]. None of the confidence intervals include zero. Thus, H5a, H5b, and H5c are supported. Human–AI collaboration event novelty has a significant negative indirect effect on information security behavior through cognitive overload. The indirect effect is −0.084, with a 95% bootstrap confidence interval of [−0.142, −0.030]. Human–AI collaboration event disruption has a significant negative indirect effect on information security behavior through cognitive overload. The indirect effect is −0.081, with a 95% bootstrap confidence interval of [−0.138, −0.028]. Human–AI collaboration event criticality also has a significant negative indirect effect on information security behavior through cognitive overload. The indirect effect is −0.076, with a 95% bootstrap confidence interval of [−0.130, −0.026]. None of the confidence intervals include zero. Thus, H6a, H6b, and H6c are supported.

**Table 7:** Results of indirect effect tests.

Hypothesis	Path	Indirect effect	Boot SE	LLCI	ULCI	Result
H5a	HACN → SV → ISB	0.034	0.015	0.009	0.068	Supported
H5b	HACD → SV → ISB	0.035	0.016	0.009	0.070	Supported
H5c	HACC → SV → ISB	0.044	0.018	0.015	0.084	Supported
H6a	HACN → CO → ISB	−0.084	0.028	−0.142	−0.030	Supported
H6b	HACD → CO → ISB	−0.081	0.028	−0.138	−0.028	Supported
H6c	HACC → CO → ISB	−0.076	0.027	−0.130	−0.026	Supported

*N* = 351. Indirect effects are standardized estimates. Boot SE denotes the bootstrap standard error. LLCI and ULCI denote the lower and upper bounds of the 95% bootstrap confidence interval, respectively. Bootstrap resampling is conducted 5,000 times.

## 5 Discussion

### 5.1 Theoretical contributions

This study makes three theoretical contributions. First, this study introduces event system theory into the context of human–AI collaborative learning among university students and provides a new theoretical perspective for explaining the behavioral consequences of artificial intelligence supported learning. As artificial intelligence becomes increasingly integrated into higher education, prior studies examine its potential benefits and risks in relation to instructional support, learning management, personalized learning, intelligent tutoring, and learning assessment (Chiu, 2024; Crompton and Burke, 2023; Zhuang et al., 2025). Bond et al. (2024) further argue that research on artificial intelligence in higher education needs to devote greater attention to ethical concerns, collaborative relationships, and methodological rigor. Recent research on generative artificial intelligence also examines student perceptions of potential benefits and risks (Chan and Hu, 2023; Chiu, 2024) as well as the collaborative relationship between humans and artificial intelligence (Atchley et al., 2024; Hwang and Lee, 2025). However, prior research largely focuses on technological functions, perceptions of use, and collaborative experiences. Limited attention is directed toward the event attributes associated with the incorporation of artificial intelligence into learning processes. Event system theory suggests that event novelty, event disruption, and event criticality jointly determine event strength and shape cognitive and behavioral responses to external changes (Morgeson et al., 2015). Drawing on this perspective, the present study characterizes human–AI collaboration event strength in terms of event novelty, event disruption, and event criticality. Accordingly, this shifts the focus of artificial intelligence supported learning research from technology use to the event attributes of human–AI collaboration and complements existing explanations for variations in individuals' cognitive and behavioral responses.

Second, this study reveals two distinct mechanisms through which human–AI collaboration event strength influences information security behavior and deepens understanding of the complex consequences of artificial intelligence supported learning. Prior research shows that artificial intelligence can improve information access and task efficiency while also generating potential cognitive risks (Farrokhnia et al., 2024). Lim et al. (2023) argue that generative artificial intelligence can simultaneously bring benefits and risks to education, indicating that its effects are not unidirectional. Zhai et al. (2024) further find that over-reliance on artificial intelligence dialogue systems may undermine students' decision making, critical thinking, and analytical reasoning. Hwang and Lee (2025) find that, during human–AI collaborative content creation, students recognize the benefits of generative artificial intelligence for idea generation and efficiency while also attending to its accuracy, limitations, and ethical concerns and continuing to revise and refine artificial intelligence outputs. Wang and Zhang (2026) further find that human–generative artificial intelligence pedagogical partnerships can simultaneously activate cognitive vigilance and cognitive offloading, which influence transformative learning through different cognitive pathways. Therefore, when artificial intelligence is incorporated into learning processes, risk recognition and cognitive demands may coexist. By considering both security vigilance and cognitive overload, this study provides a more nuanced perspective for explaining the behavioral consequences of human–AI collaborative learning.

Third, this study extends research on information security behavior to human–AI collaborative learning contexts and enriches understanding of its antecedents. Prior research primarily examines information security behavior in conventional online contexts, with particular attention to threat perceptions, coping capacity, and protection motivation (Baltuttis et al., 2024; YChen et al., 2021; Gillam and Foster, 2020). Tsai et al. (2016) find that individuals' perceptions of online threats and their responses to those threats are closely associated with online safety behavior. van Bavel et al. (2019) further show that risk prompts and response information can encourage safer online behavior. Tang et al. (2021) find that risk- and coping-related cognitive factors influence information security behavior in the context of online fraud related to the COVID-19 pandemic. These studies indicate that prior research on information security behavior has primarily explained behavioral differences through individuals' perceptions of security risks and their responses to those risks. Compared with conventional online security contexts, information security risks in human–AI collaborative learning are more closely embedded in routine learning activities and continuing interactions (Chan and Hu, 2023; Hwang and Lee, 2025). When artificial intelligence is used for learning tasks, students may exhibit different cognitive and behavioral responses. Therefore, this study incorporates the event attributes of human–AI collaboration into the antecedent analysis of information security behavior, thereby complementing existing perspectives that primarily explain information security behavior in terms of risk perceptions and coping responses.

### 5.2 Practical implications

First, universities should integrate information security education into AI literacy programs through structured courses and training initiatives. Artificial intelligence literacy involves the ability to use technological tools as well as the capacity to evaluate outputs and make appropriate ethical judgments (Almatrafi et al., 2024; Ng et al., 2021). Universities can incorporate AI security literacy content into orientation programs, general education courses, or specialized training sessions to help students identify AI-generated misinformation, evaluate source credibility, and recognize the data leakage, privacy disclosure, and cybersecurity risks associated with AI tool use. Case-based teaching and scenario-based exercises can also be used to help students understand these risks and their potential consequences. In addition, clear and accessible guidelines should be developed for different academic tasks, such as course papers, research reports, and graduation projects, specifying the appropriate boundaries of AI use, citation requirements, and verification responsibilities.

Second, instructors should optimize the design of AI-assisted learning tasks to reduce unnecessary cognitive load while maintaining appropriate security vigilance. When designing tasks that involve AI tools, instructors can provide structured verification steps or checklists to guide students through basic checks of information sources, content accuracy, and potential risks before they act upon AI-generated outputs. For more complex collaborative tasks, these assignments can be divided into stages with clear requirements and evaluation criteria, reducing the additional cognitive burden of simultaneously managing content generation, result verification, and security judgment. Universities can also offer faculty development programs on AI-assisted instruction to enhance instructors' competencies in task design, prompt engineering, and evaluation of AI-generated content, thereby maintaining necessary information security safeguards while improving learning efficiency.

Third, higher education policymakers and educational administrators should refine institutional guidelines for the safe and responsible use of AI, and promote collaborative governance mechanisms among universities, AI platform providers, and relevant technical departments. These guidelines can specify basic requirements for personal data protection, AI-generated content verification, source identification, and AI use in important academic tasks, while establishing differentiated risk management requirements for different scenarios, such as routine learning, course assignments, research training, and graduation projects. Policymakers can also encourage AI platforms to provide contextual risk notifications during student use, such as concise and actionable reminders when uploading personal data, accessing external links, or downloading unverified files, along with source labels, privacy notices, and accessible verification tools. These security requirements and notifications should avoid unnecessary complexity to prevent additional information processing demands from increasing students' cognitive load.

### 5.3 Limitations and future research

This study has several limitations that provide directions for future research.

First, the sample is drawn from students at a single university in Shanghai. The reliance on a single institution limits the geographic and institutional coverage of the sample and may therefore constrain the generalizability of the findings to other universities and regions. Students from different regions, types of universities, and fields of study may differ in their experience with artificial intelligence, the characteristics of their learning tasks, and their awareness of information security. Future research can include students from a broader range of institutions and regions and compare differences across groups to improve the applicability of the findings.

Second, a three-wave time-lagged survey design is adopted to reduce potential common method bias associated with data collected at a single time point. However, the data are still primarily derived from self-reports. Therefore, the findings may still be subject to self-report bias. In addition, because the questionnaires were distributed through internal university channels, the recruitment and data collection procedures may have introduced some degree of sample selection and data collection bias. Future research can incorporate experiments, scenario-based simulations, and platform behavior data to examine how students respond when they encounter unknown links, disclose personal information, or evaluate artificial intelligence-generated content.

Third, this study focuses on the roles of security vigilance and cognitive overload in the relationship between human–AI collaboration event strength and information security behavior. Other individual and contextual factors not included in the present study, such as AI literacy, prior information security experience, personality traits, task complexity, and platform characteristics, may also shape students' information security behavior and their responses to human–AI collaboration events. Future research can incorporate these factors and further examine relevant boundary conditions and different forms of cognitive load to provide a more detailed explanation of information security behavior in human–AI collaborative learning contexts.

## 6 Conclusion

The widespread use of artificial intelligence in higher education learning contexts is changing how university students acquire information, complete tasks, and process learning materials. Drawing on event system theory, this study uses a three-wave time-lagged survey of students at a university in Shanghai to examine how human–AI collaboration event strength influences information security behavior.

The findings show that event novelty, event disruption, and event criticality increase attention to potential security concerns while also increasing information processing pressure. Security vigilance encourages more cautious information security behavior, whereas cognitive overload reduces the capacity to respond appropriately to potential risks. The indirect effect analysis further shows that these event strength attributes are associated with information security behavior through two pathways with opposing directions.

These findings indicate that artificial intelligence supported learning involves both technological support and additional cognitive demands. Students need to evaluate information, verify content, and integrate task requirements while using artificial intelligence. Appropriate use of artificial intelligence in higher education therefore requires information security awareness as well as clear, accessible, and practical support. Such measures can help ensure that artificial intelligence contributes to learning without creating unnecessary risks.

## Data Availability

The raw data supporting the conclusions of this article will be made available by the authors, without undue reservation.

## Appendix A

Human–AI Collaboration Event Novelty

1. The way I complete learning tasks in collaboration with AI is novel to me.
2. The solutions provided by AI often surprise me.
3. The process of interacting with AI is quite different from my previous learning experience.
4. The content generated by AI often brings me unexpected information or perspectives.

Human–AI Collaboration Event Disruption

1. The involvement of AI changes my original learning process.
2. After collaborating with AI, I need to readjust my information processing methods.
3. The use of AI makes my previously accustomed learning approaches no longer fully applicable.

Human–AI Collaboration Event Criticality

1. Learning tasks completed in collaboration with AI have an important impact on my course grades.
2. The tasks I complete using AI are usually closely related to my academic goals.
3. Improper use of AI may have a significant impact on my learning outcomes.
4. I consider tasks completed in collaboration with AI to be important to me.

Security Vigilance

1. I critically evaluate the accuracy of content generated by AI.
2. I actively question the reasoning logic behind AI outputs.
3. I verify information generated by AI through independent sources. pagebreak
4. I maintain a skeptical attitude toward AI-generated responses.
5. I carefully check AI-generated content for potential errors.
6. I carefully review AI-generated content before accepting it.

Cognitive Overload

1. The topic of the learning task is very complex.
2. The formulas involved in the learning task are very complex.
3. The concepts and definitions involved in the learning task are very complex.
4. The instructions or explanations in the learning task are very unclear.
5. The instructions or explanations in the learning task are very inefficient for learning.
6. The instructions or explanations in the learning task contain many unclear expressions.
7. The learning task has genuinely enhanced my understanding of the relevant topic.
8. The learning task has genuinely enhanced my knowledge and understanding.
9. The learning task has genuinely enhanced my understanding of the relevant formulas.
10. The learning task has genuinely enhanced my understanding of the concepts and definitions.

Information Security Behavior

1. I expect that I will think twice before clicking unknown links recommended by generative artificial intelligence or downloading unknown files.
2. I expect that I will think twice before sharing personal information (e.g., student ID, contact information) with unfamiliar AI platforms.
3. I expect that I will think twice before directly using AI-generated content for assignments or reports without verification.
